# HPV infection and vaginal microecological disorders in women with intrauterine adhesion: cross-sectional study in a Chinese population

**DOI:** 10.1186/s12879-023-08659-1

**Published:** 2023-11-27

**Authors:** Li Wang, Jiuzhi Zeng, Hanbo Liu, Hongzhou Xu, Yan Liu, Mengjun Luo, Weixin Liu

**Affiliations:** 1 Reproductive Medicine Center, Key Laboratory of Reproductive Medicine, Sichuan Provincial Maternity and Child Health Care Hospital, The Affiliated Women’s and Children’s Hospital of Chengdu Medical College, 610045 Chengdu, Sichuan China; 2 Department of Clinical Laboratory, School of Medicine, Chengdu Women’s and Children’s Central Hospital, University of Electronic Science and Technology of China, No.1617 Ri Yue Street, 611731 Chengdu, Sichuan China; 3 Department of Gynecology, School of Medicine, Chengdu Women’s and Children’s Central Hospital, University of Electronic Science and Technology of China, No.1617 Ri Yue Street, 611731 Chengdu, Sichuan China

**Keywords:** IUA, Human papillomavirus, Vaginal microecological

## Abstract

**Background:**

The purpose of this study was to evaluate the vaginal microecology and the distribution of human papillomavirus (HPV) subtypes in patients with uterine adhesions and explore the correlation between HPV infection and vaginal microecology imbalance and the occurrence of intrauterine adhesion (IUA).

**Methods:**

A total of 479 women were enrolled in the study, including 259 in the normal group and 220 in the IUA group. Vaginal microecological and HPV analyses were performed on all participants. Significant differences between the two groups were analyzed, and Spearman correlation analysis was performed.

**Results:**

The incidence of IUA in patients between 31 and 40 years of age was high. The I-II degree of vaginal cleanliness in the IUA group was significantly lower than that in the normal group, and the number of III-IV degree was significantly higher than that in the normal group. Moreover, the incidences of VVC (vulvovaginal candidiasis) and vaginal disorders and infections with HPV 16 and HPV 52 subtypes were significantly higher in the IUA group than in the normal group. The incidence of high-risk HPV infection combined with vaginal disorders in the IUA group was higher than that in the normal group. Correlation analysis showed that the occurrence of IUAs was positively correlated with HPV infection and negatively correlated with PH and vaginal microecological imbalance.

**Conclusion:**

The HPV infection rate and vaginal microecology disorders affect the occurrence of IUAs. For patients with IUAs, control of the HPV infection rate and the prevention of vaginal microecological disorders should be improved.

## Introduction

Intrauterine adhesion (IUA), as known as AsHerman’s syndrome, is a group of diseases characterized by endometrial basal layer damage caused by injury to the basal layer of the endometrium of the uterine cavity or cervical canal caused by uterine cavity operation, infection, radiation and other causes, which causes part of the anterior and posterior walls or the entire walls of the uterine cavity to exhibit adhesion and fibrosis [1]. Its clinical manifestations are such as amenorrhea, recurrent miscarriage, premature delivery and difficulty in embryo implantation, are also diverse. In clinical practice, approximately 30–50% of women who failed in early pregnancy received surgical treatment because of incomplete evacuation in the world. At the same time, in terminating a pregnancy, when fragments of the pregnancy remain in the uterine cavity, uterine curettage is the most common procedure. IUA is the most common complication of curettage and the incidence of IUA after uterine curettage is between 15% and 40%. Hysteroscopy also revealed a 19.1% incidence of IUA within one year of medical and surgical treatment in women [2, 3]. In recent years, this condition has attracted wide clinical attention because it seriously affects the fertility of women of childbearing age.

Previous studies have also reported a significant correlation between vaginal pH and human papillomavirus (HPV) infection, especially in women younger than 35 [4]. HPV is generally a nonlytic infection, and the inflammatory response generated by HPV is slightly less severe. However, after mucosal infection, such as with chlamydia and bacteria, the inflammatory response and altered tissue features could lead to IUA [5, 6]. The exact mechanism underlying the effects of beneficial vaginal bacteria and HPV and its relationship with infection and inflammation still need more research. Recent studies have shown that bacteria in the vagina can affect vaginal cytokines and chemokines, including Interleukin 18 (IL-8) and Interleukin (IL-2β), and bacterial activation of the production of the proinflammatory transcription factors NF-γB and tumor necrosis alpha (TNF-α) can occur [7–9]. Bacterial vaginosis (BV) induced vaginal microecology disorder is the most common vaginal infection among women of childbearing age, and it is also the disease most closely related to HPV infection. Relevant studies have confirmed that it can increase the incidence rate of HPV infection [10]. HPV infection, especially high-risk HPV (HR-HPV) infection, has attracted widespread attention because of its close relationship with cervical cancer [11]. The HPV infection rate among Chinese women was 15.71%, and 84.6% of sexually active women were found to have been infected with HPV at least once in their lifetime [12]. Our previous studies have confirmed that the incidence of HPV is significantly increased in patients with infertility and has a considerable correlation with infertility [13].

In recent years, most studies on mycoplasma, chlamydia infection, vaginal microecology and HPV infection have focused on the relationship between the above and cervical diseases [14]. At present, the prevention of HPV infection still needs considerably more progress, but the timely detection and blocking of HPV infection are still the main strategies used for prevention [15]. However, whether there is a relationship between HPV infection and IUA and the incidence of HPV infection and vaginal microecology imbalance in patients with IUA has not been reported thus far. We conducted a cross-sectional study based on the relationship between IUA and the abovementioned factors in Western China. This study expected to provide value as a reference for the prevention and treatment of IUAs.

## Materials and methods

### Study population

#### Data collection

Participants were recruited by clinicians and underwent hysteroscopy in the Department of Gynecology of Chengdu Women’s and Children’s Central Hospital, School of Medicine, University of Electronic Science and Technology of China, from January 2020 to October 2022. The patients were enrolled in the group according to the references [16], and hysteroscopy was performed on the women who complained of oligomenorrhea, amenorrhea and infertility. All patients were examined within 3–7 days of undergoing nature hysteroscopy after menstruation. A total of 220 patients diagnosed with IUAs were included in the IUA group. A total of 259 women with normal uterine cavities aged 18–50 years were selected as the normal group and hysteroscopy ruled out IUAs. The diagnostic criteria of IUA refer to the scoring criteria of the American Fertility Society (AFS) and European Society of Gynecological Endoscopy (ESGE). The exclusion criteria were as follows: (1) treatment with topical vaginal medication within 1 month and use of estrogen and progesterone drugs; (2) immune diseases or malignant tumors; and (3) endometrial polyps, uterine fibroids, adenomyosis and other reproductive systemic diseases. All vaginal and cervical swab samples were collected by a professional doctor according to the method described in the reference [13]. Before hysteroscopic surgery, the cervix was exposed, and then a sufficient epithelial cells were collected with a cervical brush and placed in the eluent for HPV detection. Vaginal secretion samples were collected from the posterior vaginal fornix and two vaginal swabs were collected with aseptic swabs to determine the pH value and the concentration of hydrogen peroxide. All procedures and protocols applied in this study were approved by the ethics committee of the Women and Children Affiliated Hospital of the Medical College of the University of Electronic Science and Technology (Grant No. B2019(1)), and informed consent was obtained from all subjects.

### Microecological detection

Vaginal smears were subjected to Gram staining [17]. After Gram staining, vaginal cleanliness, white blood cells, Lactobacillus number, Candida spp. (spores, blastospores, and pseudohyphae), Trichomonas vaginalis, clue cells, the bacterial density, the flora diversity and the dominant bacteria were observed. The observations followed the guidelines of the National System for External Quality Assessment (NSEQA) and College of American Pathologists (CAP) [18].

The diagnostic criteria of the bacterial density, vaginal health, trichomonal vaginitis (TV), vulvovaginal candidiasis (VVC) and bacterial vaginosis (BV) were carried out according to the criteria of references [13, 19, 20]. Bacterial density refers to the witness and distribution of bacteria under a microscope, which is divided into four grades according to the density observed under the microscope: grade I (1 +): 1–9; grade II (2 +): 10–99; grade III (3 +): 100 and above; and grade IV (4 +): bacterial clusters in the full visual field. Flora diversity refers to the number of all types of bacteria in the microscope field of view under high magnification (1000 times). The diversity of vaginal flora was divided into four grades: grade I (1 +): 1–3 flora species; grade II (2 +): 4–6 flora species; grade III (3 +): 7–10 flora species; and grade IV (4 +): more than 10 flora species. According to the Expert Consensus on the Clinical Application of Vaginal Microecosystem Assessment of the Cooperative Group of Infectious Diseases [21], the normal vaginal microecosystem is defined as vaginal health grade I, bacterial density grades II-III, flora diversity grades II-III, and Lactobacillus as the dominant bacterial species. In addition, the vaginal pH value was 3.8–4.5, and the production of hydrogen peroxide was normal. When any of the above indicators (such as vaginal health, bacterial density, bacterial diversity, dominant bacteria, pH or lactobacillus abundance) are abnormal, microecological disorders are diagnosed.

### HPV genotyping detection

The HPV genotyping test uses a diagnostic kit for HPV detection to detect 24 HPV genotypes, including high-risk HPV subtypes 16, 18, 31, 33, 35, 39, 45, 51, 52, 53, 56, 58, 59, 66, 68, 73 and 82 and low-risk subtypes 6, 11, 42, 43, 81 and 83. DNA was extracted using the corresponding reagent kits. Nucleic acid amplification and reaction of DNA samples were performed using a Roche Lightcycler 480 nucleic detector. The reaction consisted of 2 μl of DNA, 27.6 μl of HPV PCR mix (including specific primers), and 0.4 μl of Taq/UNG (final volume: 30 μl). The PCR conditions were 1 cycle of hot-start activation at 95 °C for 10 min; followed by 40 cycles of denaturation at 95 °C for 30 s, annealing at 52 °C for 30 s, and extension at 65 °C for 30 s; and a final extension at 65 °C for 5 min. Then, the amplified product was specifically amplified again by an HPV gene chip. The chip detection and reading system was used to scan and analyze the hybridized chip image and finally obtain the HPV genotyping results.

### Statistical analysis

All data were analyzed with SPSS statistical software SPSS 22.0 (IBM, Chicago, USA). The χ2 test was used to analyse the differences in microecological factors and HPV detection proportions between groups. Spearman correlation coefficients were computed to examine the associatiion between HPV and vaginal microbiome and IUA. P < 0.050 indicated that the difference was statistically significant.

 .

## Results

### Study population

Table 1 shows general data for 479 patients, including 220 in the IUA group and 259 in the normal group, who ranged in age from 18 to 50 years old. We grouped the subjects according to their age. To our surprise, there was no significant difference in the incidence of IUA of patients between 18 and 30 years. Patients 31–40 years of age had a significantly higher incidence of IUA than the normal group, and in patients between 41 and 50 years of age, there was a significant difference between the two groups (Table 1).

**Table 1:** Baseline characteristics of the 479 women

Variables	IUA group (n = 220) Case (%)	Normal group (n = 259) Case (%)	*χ* ^*2*^	*P*
Age (years)	32.24 ± 4.80	33.24 ± 7.76		*0.084*
18–30 (years)	85 (38.64)	111 (42.86)	*0.877*	*0.349*
31–40 (years)	126 (57.27)	100 (38.61)	*16.626*	*< 0.001*
41–50 (years)	9 (4.09)	48 (18.53)	*23.666*	*< 0.001*

### Vaginal microecology analysis of the IUA and normal groups

There was no significant difference in vaginal pH between the IUA and normal groups. The normal group had more patients with vaginal cleanliness I-II than in the IUA group, and the III-IV degree was less than that in the IUA group, and the degree of III-IV was less than that in the IUA group. The incidence of vulvovaginal candidiasis (VVC) was higher in the IUA group than in the normal group. Correspondingly, there was no significant difference between the two groups in the rates of BV and trichomonas vaginitis (TV) or in the vaginal microecology (Table 2).

**Table 2:** Analysis of differences in pathogenic microorganisms and vaginal microecology in the IUA and normal groups

Variables	IUA group (n = 220) Case (%)	Normal group (n = 259) Case (%)	*χ* ^*2*^	*P*
PH	4.86 ± 0.447	4.81 ± 0.489		*0.211*
I-II	136 (61.81)	193 (74.52)	*8.919*	*0.003*
III-IV	84 (38.18)	66 (25.48)	*8.919*	*0.003*
BV	148 (67.27)	166 (64.09)	*0.533*	*0.465*
VVC	16 (7.27)	8 (3.09)	*14.510*	*< 0.001*
TV	0 (0)	1 (0.39)	*0.851*	*0.356*
Vaginal dysbiosis	168 (76.36)	199 (76.83)	*0.015*	*0.904*

Trichomonas vaginitis (TV); vulvovaginal candidiasis (VVC); bacterial vaginosis (BV).

### Distribution of HPV subtypes in the IUA and normal groups

There was no significant difference in the prevalence of infections with low-risk HPV types, including HPV6, HPV11, HPV42, HPV44, HPV81 and HPV83, between the IUA group and normal groups. The incidence of infections with high-risk HPV16 and HPV52 was significantly higher in the IUA group than in the normal group. There was no significant difference between the two groups regarding infections with the high-risk HPV types, including HPV 18, 31, 33, 35, 39, 45, 51, 53, 56, 58, 59, 66, 68, 73 and 82 (Table 3).

**Table 3:** Differences in the distribution of HPV subtypes in IUA and normal groups

Infection types	HPV genotype	IUA group Infection case	Normal group Infection case	*χ* ^*2*^	*P*
Low risk	HPV6	3	2	*0.403*	*0.526*
	HPV11	5	1	*0.403*	*0.526*
	HPV42	0	1	*0.851*	*0.356*
	HPV44	2	0	*2.273*	*0.132*
	HPV81	0	0		
	HPV83	0	0		
High risk	HPV16	8	2	*4.774*	*0.029*
	HPV18	2	2	*0.027*	*0.870*
	HPV31	2	0	*2.364*	*0.124*
	HPV33	1	1	*0.013*	*0.908*
	HPV35	1	1	*0.013*	*0.908*
	HPV39	2	0	*2.364*	*0.124*
	HPV45	0	0		
	HPV51	3	1	*1.373*	*0.241*
	HPV52	9	2	*5.762*	*0.016*
	HPV53	2	1	*0.523*	*0.470*
	HPV56	3	3	*0.041*	*0.840*
	HPV58	3	2	*0.403*	*0.526*
	HPV59	1	1	*0.013*	*0.908*
	HPV66	1	1	*0.013*	*0.908*
	HPV68	0	0		
	HPV73	1	0	*1.180*	*0.277*
	HPV82	0	0		

### HPV infection in individuals of different ages in the IUA and normal groups

Our study revealed that the prevalence of HPV was significantly higher in the IUA group than in the normal group. We further analyzed the incidence of HPV infection in the IUA group and the normal group of all ages, and we found that there was no significant difference between the two groups in patients aged 18–30 years. The incidence was significantly higher in patients between the ages of 31–40 and 41–50 years (Table 4).

**Table 4:** Differences in HPV infection between the IUA and normal groups

Variables	IUA group			Normal group		*χ* ^*2*^	*P*
	n	HPV Infections cases (%)		*n*	HPV Infection cases (%)		
18–30	85	10 (11.76)		111	4 (3.60)	*3.776*	*0.052*
31–40	126	22 (17.46)		100	8 (8)	*9.678*	*0.002*
41–50	9	0 (0)		48	6 (12.5)	*5.161*	*0.023*
Total	220	32 (14.55)		259	18 (6.95)	*7.341*	*0.007*

### The association between the vaginal microbiome and HPV infection in the IUA and normal groups

We analyzed the association between high-risk and low-risk HPV infection and vaginal microecological dysbiosis and normal vaginal microecology in the IUA group and normal group (Table 5). We found that the incidence of high-risk HPV infection with vaginal dysbiosis in the IUA group was significantly higher than that in the normal group, but there was no significant difference in the low-risk type. There was no difference in infection between the low-risk and high-risk HPV groups with normal vaginal microbiota (Table 5).

**Table 5:** Vaginal microbiome and HPV infection proportions in the IUA and normal groups

Vaginal microecology		IUA group		Normal group	*χ* ^*2*^	*P*
		Infection case (%)		Infection case (%)		
Vaginal dysbiosis	Low risk	6 (3.57)		5 (2.51)	*0.264*	*0.608*
	High risk	22 (14.01)		8 (4.02)	*8.986*	*0.003*
	total	28 (16.67)		13 (6.53)	*8.250*	*0.004*
Normal Vaginal microecology	Low risk	2 (3.85)		0 (0)	*2.273*	*0.132*
	High risk	8 (15.38)		5 (2.51)	*1.149*	*0.284*
	total	10 (19.23)		5 (2.51)	*2.429*	*0.119*

### Correlation analysis

The incidence of IUAs was positively correlated with the HPV infection rate (r = 0.123, p = 0.007) and negatively correlated with vaginal pH (r=-0.098, p = 0.031) and vaginal microecological imbalance (r=-0.209, p < 0.001).

## Discussion

In recent years, the incidence of IUA has gradually increased among women who have experienced various miscarriages and curettages, affecting 6–30% of women of childbearing age [22, 23]. After the operation, even if the uterine cavity can be restored, the endometrium cannot be completely repaired. The impact of IUA on women’s fertility is enormous. Therefore, effective prevention after IUA is important [24]. The main cause of IUA is serious destruction of the integrity of the endometrium, which causes the surfaces of the uterine wall to fuse with each other. During the process of fusion, limiting inflammation is vitally important. Therefore, the environment of the uterine cavity is an important condition to limit inflammation [25, 26]. In the pathogenesis of IUAs, the changes in the uterine microenvironment are a factor, and the potential contribution of the bacterial microbiota and pathogen infection of the reproductive tract to female reproductive health may promote the occurrence of endometrial polyps, and infertility following uterine adhesion [13]. Previous studies have reported that changes in the bacterial microflora of the reproductive tract and infections with pathogenic bacteria such as *Mycobacterium tuberculosis* and *Candida* infection can promote the occurrence of IUA [25]. In our study, we also found that vaginal microecological changes in patients with IUAs, the degree of vaginal cleanliness and the infection rate of VVC in patients with IUAs were significantly different from those in the normal group. This is consistent with previous literature. Vaginal cleanliness imbalance, to a certain extent, will induce vaginitis, leading to flora imbalance, thus creating conditions for the occurrence of IUAs. *Candida albicans* infection is significantly related to the degree of inflammation and fibrosis in IUA, and is an independent risk factor for the occurrence of IUA [16, 27].

The reproductive tract of healthy women is where a variety of microorganisms coexist, and maintaining a good balance and resisting the invasion of microorganisms play important roles in maintaining the health of the reproductive tract [28]. In our study, we found that IUAs occurred more frequently in women aged 31–40 and 41–50 years. This is consistent with the infection rate of HPV in IUA patients. Although there are lower fertility requirements of women aged 41–50 years and there are fewer patients who undergo further hysteroscopy, the occurrence of IUA was consistent with HPV infection. Previous studies have reported that HPV infection has a significant correlation with age [29]. Age and other risk factors can also increase the incidence of IUA [30]. Although HPV vaccination has been increasingly confirmed to have a preventive and adjuvant treatment effect on cervical cancer and although HPV screening and prevention strategies for HPV are constantly being strengthened, HPV infection still has a high incidence [31, 32].The latest research shows that inflammation and oxidative stress play are indispensable factors in the pathogenesis of IUA. In the process of chronic inflammation, HPV can interfere with the dynamic redox balance of host cells, further influencing oxidative stress, thus promoting the persistent release of many molecules from inflammatory cells, resulting in cell damage, and the most significant types of HPV are HPV16 and HPV52 [33, 34]. In our study, the infection rates of high-risk HPV16 and HPV 52 in the IUA group were significantly higher than those in the normal groups, and the above results suggest that HPV may promote the pathogenesis of IUA.

Postoperative recurrence of IUA is common in the clinic, and the interaction between IUA and the vaginal microbiome may have a great impact on microbiome diversity, especially the number of *Lactobacillus* and *Gardnerella* vaginalis in patients with IUA. In patients with IUA, it was observed that the number of *Lactobacillus* decreased significantly, while there was overgrowth of pathogenic *Gardnerella* and *Prevotella* [30]. Considering the relationship between inflammation, fibrosis and vaginal microecology, we further explored the relationship between vaginal microecological disorders and HPV infection and IUAs. First, we analyzed patients with vaginal microecology disorder and HPV infection and found that the incidence of high-risk HPV infection and vaginal microecological disorders was also significantly higher in the IUA group. HPV infection and vaginal microbial dysbiosis are positively correlated with the occurrence of IUAs. This is consistent with the direct relationship between vaginal microecological disturbance and HPV infection and infertility, cervical cancer, and maternal and neonatal outcomes of pregnant women [13, 35]. Our study confirmed for the first time the relationship between HPV and vaginal microbial dysbiosis and the occurrence of IUAs. The occurrence of IUAs is significantly negatively correlated with vaginal pH and vaginal microbiota imbalance and positively correlated with HPV infection. The vaginal microecology has a regulatory effect on IUAs. Appropriately increasing the number of *Lactobacillus* in the vagina may reduce the recurrence rate of IUA to a certain extent [30]. However, our study requires more in-depth research due to the sample size and to determine its exact mechanism of action. However, our study still suggests that for IUA patients, paying attention to vaginal microecology and prevention of HPV infection may play an important role in reducing IUA recurrence and enabling the prevention of IUA.

The pathological changes of IUA will inevitably affect the microbiota and metabolites in the uterine cavity, thereby affecting the diversity of the vaginal microbiota. The cascade reaction caused by HPV infection promotes inflammation and is also highly involved in the disease process of IUA. The combined reaction caused by changes in the vaginal flora and from HPV infection jointly promotes the progression and recurrence of IUAs. Although our study has clarified the correlation between IUA and vaginal microbiology and HPV to a certain extent, we need more in-depth large-sample studies to further explore the exact regulatory mechanism and provide guidance for the clinical treatment and prevention of IUA.

## Conclusion

Our study shows that vaginal microecological disorders and HPV infection may play a role in the occurrence of IUAs. The subtypes of HPV and the imbalance of vaginal microecology are directly related to IUAs. Our study explored the HPV infection and vaginal microecology of patients with IUAs at different ages. However, due to the limitations of the sample size and detection methods, and lack of data on mechanism. The study of the effects of vaginal microecology and HPV on IUAs needs further prospective research.

## Data Availability

The datasets used or analyzed during the current study are available from the corresponding author upon reasonable request. All data are included in the paper and references.
